# Supplementary material for: Regulatory Resistance? Narratives and Uses of Evidence around “Black Market” Provision of Gambling during the British Gambling Act Review
Source: Int J Environ Res Public Health. 2021 Nov 3;18(21):11566. doi: 10.3390/ijerph182111566 (PMC8582964; doi:10.3390/ijerph182111566)
Supplement: Supplementary file 1 [file ijerph-18-11566-s001.zip › ijerph-1400931-supplementary.pdf]

Supplementary Material: Regulatory resistance? Narratives and uses of evidence around "black market" provision of gambling during the British Gambling Act Review

Search Protocol

**Research question**	What do recent media reports tell us about how narratives around the black market for gambling are being framed, and by whom?
**Methods**	
**Search databases:**	Google News; Lexus Nexis
**Search terms (in title/abstract)**	Gambling AND "black market" OR "unlicensed" OR "unregulated" OR "illegal" OR "illicit" OR "underground"
**Inclusion criteria:**	
**Types of articles**	Any UK news article of gambling which includes any mention of the black market/unlicensed gambling/unregulated gambling in Britain.<br><br>To include journalist reports, op-eds, comments and letters to editors
**Dates**	Published between 8th December 2020 and 26th May
**Other**	UK only
**Data collection and analysis:**	
**Selection of studies**	Searches will be conducted and screened according to the inclusion criteria by the review author. The full text of any potentially relevant articles will be retrieved for closer examination. All articles which initially appear to meet the inclusion criteria but on inspection of the full text paper do not meet the inclusion criteria will be detailed in a table 'Characteristics of excluded studies' together with reasons for their exclusion. The results of the study selection process will be presented in a PRISMA format flow chart.
**Data extraction**	Data extracted for each article will include:<br>• Publication<br>• Title of article

	• Date of article<br>• Author/By-line (if appropriate)<br>• Type of articles (journalist report/op-ed/comment/letter to editor)<br>• Main focus of article (black market; other changes to gambling regulation; gambling policy etc)<br>• Summary of black market discussion<br>• Who is commenting on black market (if appropriate)<br>• Context of comment on black market (in response to regulatory change; in response to conversation about economy etc)<br>• Whether comments on black market are contextualised or critiqued
**Data synthesis**	Descriptive summaries about the articles published will be generated. Narrative synthesis about ways in which arguments around the black market in Britain are framed.
